# Sustained Release T3 Therapy: Animal Models and Translational Applications

**DOI:** 10.3389/fendo.2019.00544

**Published:** 2019-08-13

**Authors:** Thaer Idrees, John D. Price, Thomas Piccariello, Antonio C. Bianco

**Affiliations:** ^1^Section of Endocrinology, Diabetes and Metabolism, University of Chicago, Chicago, IL, United States; ^2^Synthonics Inc., Blacksburg, VA, United States; ^3^Department of Chemistry, Virginia Tech, Blacksburg, VA, United States

**Keywords:** hypothyroidism, liothyronine, combination therapy, animals, thyroid, levothyroxine

## Abstract

The standard of care to treat hypothyroidism is daily administration of levo-thyroxine (LT4). This is based on the understanding that deiodinases can restore production of T3 and compensate for the small amounts of T3 that are normally produced by the thyroid gland. However, pre-clinical and clinical evidence indicating that deiodinases fall short of restoring T3 production is accumulating, opening the possibility that liothyronine (LT3) might have a role in the treatment of some hypothyroid patients. LT3 tablets taken orally result in a substantial peak of circulating T3 that is dissipated during the next several hours, which is markedly distinct from the relative stability of T3 levels in normal individuals. Thus, the effort to developing new delivery strategies for LT3, including slow release tablets, liquid formulations, use of T3-related/hybrid molecules such as T3 sulfate, poly-zinc-T3 and glucagon-T3, nanoparticles containing T3, subcutaneous implant of T3-containing matrices, and stem cells for de novo development of the thyroid gland. This article reviews these strategies, their applicability in animal models and translatability to humans.

## Introduction

Hypothyroidism is a common disorder that in most cases results from insufficient activity of the thyroid gland (1). The thyroid secretion contains both thyroxine (T4) and triiodothyronine (T3), with the latter being the active form of thyroid hormone (TH). It is estimated that healthy adult subjects produce approximately 100 ug T4 daily; the daily production rate of T3 is approximately 30 ug, of which only about 5 ug are secreted from the thyroid, with the remaining 25 ug produced via T4 deiodination outside of the thyroid parenchyma (2). During hypothyroidism, the reduction in circulating TH levels is detected by the hypothalamus-pituitary-thyroid (HPT), hence elevating serum TSH and defining the diagnosis of hypothyroidism (3). From the time it was originally described in the late 18th century until the 1970s, hypothyroidism was treated predominantly with desiccated thyroid extract, which contains both T4 and T3 (4). The discovery that humans convert T4 to T3 and the development of a TSH immunoassay that could be used to titrate the thyroid replacement dose, levothyroxine (LT4), became the standard of care and the need for supplementation with LT3 obviated; more recently combined therapy with LT4 and LT3 has been the subject of much discussion and controversy (4).

The thyroid function is activated by the pituitary hormone TSH, which is under direct hypothalamic stimulation via TRH and inhibition via a feed-back negative mediated by circulating T4 and T3, each playing independent roles (5). Plasma T3 is detected directly by the hypothalamic paraventricular nucleus, where TRH is produced, and in the pituitary thyrotrophes, where TSH is secreted. In contrast, in order to slow down expression of TRH and TSH, plasma T4 requires local conversion to T3 via the type 2 deiodinase (D2), which is present in the hypothalamus and in the anterior pituitary gland (6). The independent role of T4 is observed as serum TSH increases with the drop in serum T4 during iodine deficiency or mild hypothyroidism, while circulating T3 remains within normal range (7, 8). In turn, the acute administration of large doses of PTU to thyroidectomized individuals kept on L-T4-replacement therapy revealed how serum T3 *per se* has an important role in TSH secretion (9). The approximately 20% drop in serum T3 that follows as a result of D1 inhibition is sufficient to double serum TSH levels, even as serum T4 levels remain stable (9).

TH signaling is initiated via binding of T3 to nuclear receptors (TR) (10). Based on the affinity of TRs for T3, it is considered that normal circulating levels of T3 account for the bulk of TH signaling in target tissues (11). In some organs, however, the activity of local deiodinases can modulate TH signaling provided by plasma T3. Tissues that express D2 such as brain, pituitary gland and brown adipose tissue, have enhanced TH signaling because D2 produces T3 locally, which adds to the incoming T3 from circulation. In contrast, TH signaling is dampened in tissues expressing the type 3 deiodinase (D3), such as placenta, pancreatic beta cells, skin, which inactivates both T4 and T3 (11). Given that tissues and plasma T3 are in equilibrium, deiodinases produce most circulating T3 and play a role in maintaining circulating levels of T3 relatively stable. Notwithstanding, the thyroid gland also sustains plasma T3 levels as seen in animal models of deiodinase deficiency (12, 13). In fact, TSH acts on the thyroid by preferentially accelerating T3 secretion (over T4) (14–16). For example, the minimal circadian rhythmicity observed in plasma T3 levels is thought to result from an elevation in circulating TSH in the early morning hours [see (11) for review].

The standard of care for hypothyroidism is treatment with levothyroxine (LT4) that is adjusted based on serum TSH levels. The goal is to give patients sufficient amounts of LT4 to bring serum TSH within the normal reference range (3). However, since the early 70s it became apparent that therapy with LT4 results in significantly higher T4 and lower T3 serum levels, what was attributed to the absence of thyroidal T3 secretion (17). Nevertheless, a later study from the same group reported that LT4-treated patients have normal T3 and significantly higher T4 serum levels (18). In addition, a non-cross sectional study that looked at serum T3 levels in 50 euthyroid individuals before and after thyroid surgery found that therapy with LT4 can restore both circulating TSH and T3 levels (19). Hence the expectation that in LT4-treated patients the deiodinase system will metabolize LT4 and produce T3 in amounts equivalent to what is produced by the healthy thyroid gland. However, there is evidence indicating that this indeed might not be the case (20, 21). In a series of approximately 2,000 LT4-treated hypothyroid patients, serum T3 is relatively lower and serum T4 is relatively higher when compared to control individuals; in about 15% of the patients, serum T3 is below normal range (22). Similar findings were obtained through the analyzes of publically available NHANES data, showing that approximately 500 individuals maintained on LT4 have lower serum T3 levels when compared with control individuals matched for age, sex, ethnic background and serum TSH (23).

Preclinical studies of LT4-treated thyroidectomized rats indicate that minimally reduced plasma T3 levels are sufficient to cause widespread dampening in TH signaling, including in the liver, skeletal muscle and brain (24). There is also evidence that TH signaling might not be fully normalized in LT4-treated hypothyroid patients. For example, in association studies LT4-treated patients that have a normal serum TSH weigh about 10 pounds more despite ingesting less calories; they report less physical activity and are more likely to be on statins and anti-depressive therapy (23). A meta-analysis of LT4-treated patients revealed that both total cholesterol and LDL cholesterol remains elevated despite serum TSH that was within the reference range (25). In fact, these findings go along with the observation that non-objective symptoms of hypothyroidism remain in about 15–20% of the LT4-treated hypothyroid patients (difficulty with weight management, low energy, depressed mood, and memory impairment) despite achieving normal TSH and free T4 levels (26, 27). However, progress in this area is slow given the multiple biases which impact on subject self-reported quality of life that are not thyroid related.

**Figure 1:**
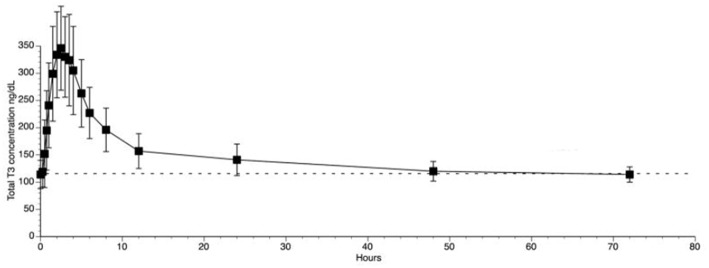
T3 serum concentrations for 3 days following oral administration of a single 50 mcg dose of liothyronine to volunteers. Adapted from Jonklaas et al. (30).

The use of liothyronine (LT3) in combination with LT4 to treat hypothyroidism is not frequent but it is popular among patients that, despite normal circulating TH and TSH, experience residual symptoms (28). However, available oral LT3 preparations result in rapid T3 absorption that is followed by fast metabolization (29). This typically results in a peak of serum T3 at about 3h after dosing, which is followed by a relatively fast decline in serum T3 levels (29–31) (Figure 1). In patients taking 10 ug of LT3, the serum T3 peak is approximately 40% above the baseline levels (32); multiple daily doses might partially mitigate this problem but its practicality is a challenge. The peak of serum T3 observed after oral administration of LT3 is not physiological and has given pause to physicians that wish to prescribe LT3. Thus, the American Thyroid Association recommends “against the routine use of combination treatment with LT4 and LT3 as a form of thyroid replacement therapy in patients with primary hypothyroidism” (3). Subsequently, in an attempt to identify adverse outcomes for patients on LT3 tablets, cardiovascular, skeleton, and mental outcomes were assessed through an observational study in the Scottish town of Tayside (33). Compared to the about 34,000 patients taking only LT4, those using LT4 plus LT3 (*n* = 327) or LT3 alone (*n* = 73) had no increased mortality or morbidity risk due to cardiovascular disease, atrial fibrillation, or fractures after adjusting for age; the number of prescriptions for bisphosphonates or statins were also similar. A novel finding was an increased risk of new prescriptions for antipsychotic medication, proportional to the number of LT3 prescriptions (33). However, prospective safety studies to evaluate potential long term undesirable cardiovascular and skeleton associated with such pharmacokinetics (PK) have not been performed (34).

### Delivery Systems That Aim at Physiological Replacement of T3

The utilization of animal models to develop systems that restore physiological levels of T3 has two main purposes. First, to study thyroid economy and TH action in other species. This is justified because hypothyroidism not only affects about 5% of the human population but is also frequently observed in other animal species such as dogs and cats (35, 36). Second, to develop delivery systems that eventually could be transitioned/adapted to human use, which has been done mostly in small laboratory rodents such as rats and mice. When studying or using animal models, however, one should be aware that thyroid economy in various species might not emulate what happens in humans (37). For example, rodents secrete relatively more T3 from the thyroid gland (about 40% of the daily production rate) and exhibit an approximately 12–24 times faster turnover rate for both serum T4 and T3 (2). However, when focusing on the absorption kinetics, rodents exhibit a similar PK profile as humans, i.e., a serum peak at 3 h after dosing that is followed by a fast decline in its circulating levels (38).

#### Enteral/Gastrointestinal Preparations

##### Oral

Administration of LT3 through the mouth can be utilized to restore TH signaling in laboratory animals that have been made hypothyroid. Adding LT3 to the drinking water or to the diet avoids the administration of a single dose of LT3 and has been shown to restore thyroid status in thyroidectomized rodents (37). However, consistency of dosing and the inevitable circadian rhythmicity associated with food and water intake remain a limiting factor. For example, LT3 supplied in the drinking water (dilution from a stock solution prepared in 40 mM NaOH) was given to different mouse strains aged 6–8 weeks, with variable results. Some strains exhibited complete (>90%) suppression of serum T4 and TSH whereas in others, a much weaker effect was observed. In addition, female and male animals responded differently, and heavier mice had higher post mortem levels of serum T3, which might have been due to increased water intake and/or changes in volume of distribution (39, 40). Notably, liquid preparations for oral administration of LT3 are available for humans (IBSA, Switzerland). Some patients might find it practical to use LT3 solutions, but few studies have been performed to date (41). Only one systematic study exists with this formulation but unfortunately PK profiles were not obtained (41).

New platforms continue to be developed to improve dosing flexibility and PK properties for oral administration of LT3. An original concept is the utilization of thermal inkjet (TIJ) 2D printing to enable the deposit of LT3 and/or LT4 onto orodispersible films (ODFs) (42). In a recent study, a two-cartridge TIJ printer was used to print separate solutions of LT3 and LT4. Dose adjustments allowed for LT3 (15–50 μg) and LT4 dosages (60–180 μg) to be successfully printed onto ODF. When placed in water, ODFs disintegrated in less than 45s (42). PK studies have not been performed but the prospect of fine customization of LT4 and LT3 dosages is exciting. Furthermore, it is conceivable that the utilization of different ODFs could effectively modify the PK properties of orally administered LT3.

An alternative approach to improve LT3 PK properties when given orally is to delay its absorption. This has been used for LT3 or combinations of LT4+LT3. For example, “slow release” LT3 tablets containing a hydrophilic swellable matrix system made with hydroxypropylmethylcellulose, sodium carboxymethylcellulose, calcium phosphate and magnesium stearate have been prepared (US Patent #5,324,522). Other combinations of salt and matrices have also been tested, including mannitol, magnesium stearate, calcium phosphate, and microporous polypropylene (US Patent #5,324,522). When tested *in vitro* the rate of LT3 release from such capsules can be modulated according to their content and grade of Methocel, and/or SimpleCap/Lactose (43). *In vivo* tests were also performed. In one of the two clinical studies performed to date, slow release LT3 tablets were given to 17 hypothyroid individuals. The results indicated that slowing down the release of LT3 in the intestine decreases the peak T3 in the serum (Cmax) by about 9% and prolonged the time to Cmax from ~3.2 to 5 h (44). However, in the other study in which LT3 tablets were prepared with microcrystalline cellulose and magnesium stearate (BCT303), sustained serum T3 levels were not observed (30). Another approach still under testing is to deliver LT3 via chewable ion exchange resin that form a drug resin complex (Spectrix, Southlake TX). This technology utilizes ion exchange resin to form a multi-particulate drug-resin complex that could potentially provide an enhanced drug release profile and PK profile in humans, but clinical studies have yet to be performed.

To meet the challenge of creating sustained and/or slow release delivery systems for LT3, investigators utilized T3 derivatives. For example, it is well-known that phenolic hydroxyl within the T3 molecule can be sulfated (T3-S), a reaction that inactivates T3 but dramatically enhances its solubility in water and loss to the environment. However, sulfatases in the liver can reactivate T3-S via de-sulfation and prevent its loss to the environment (45). Studies in hypothyroid rats revealed that substantial amounts of parentally administered T3-S were converted back to T3, triggering systemic thyromimetic effects (46). Similar studies were conducted in 28 thyroidectomized individuals given T3-S orally. Tmax for T3-S was between 3 and 4 h. At the same time, T3 levels (generated through T3-S de-sulfation) increased rapidly in the circulation, with an early peak between 2 and 4 h that was followed by a variable plateau that depended on the dose of T3-S administered and lasted up to 48 h. These observations suggest that sufficient amounts of orally administered T3-S are converted to T3 in humans and could be tested as a tool to restore T3 levels in hypothyroid individuals (47). However, studies in euthyroid individuals have not been conducted. This is important because T3-S is metabolized through the D1 pathway through inner ring deiodination, which leads to irreversible inactivation of the T3 molecule (45). In fact, D1 is expressed in such high levels in the liver and kidney that it has been proposed that D1 plays a scavenger role by minimizing the loss of iodine through urine and bile (48). This is critical given that D1 is a T3-responsive gene, hence its levels are low in tissues of hypothyroid patients. Studies could be conducted in hypothyroid individuals after thyroid status was normalized with LT4 to ensure that at the normal D1 levels there is sufficient amounts of T3-S to be de-sulfated to T3. Nevertheless, this remains an ingenious strategy that should be pursued further.

**Figure 2:**
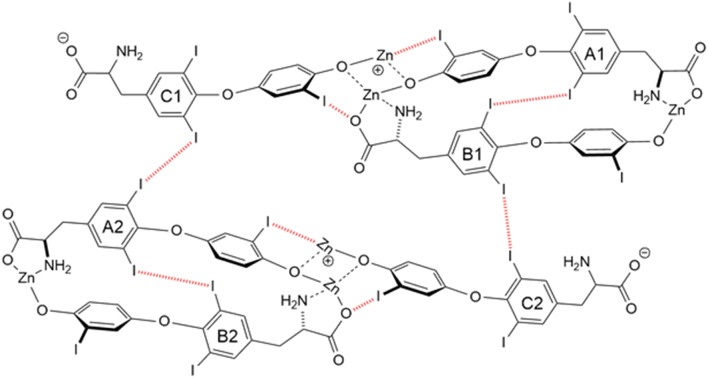
Strong bonding interactions (coordinate covalent bonds) between Zn and ligand donor atoms of T3^2-^ are shown in black; weak bonding interactions (halogen bonds) between iodine and X-bond acceptor atoms of T3^2-^ are shown in red dashed line. Both bonding modes contribute to polymer formation and stabilization. Two and 3-D structures, including metal organic frameworks, are possible.

**Figure 3:**
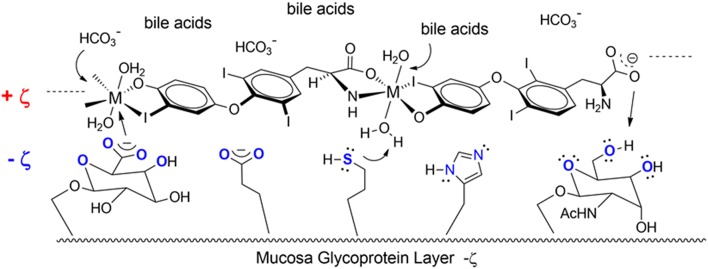
Endogenous ligands in the upper GI tract can affect hydmlysis rate. These include HCI (stomach), bile acids and carbonate buffers (upper intestines).

**Figure 4:**
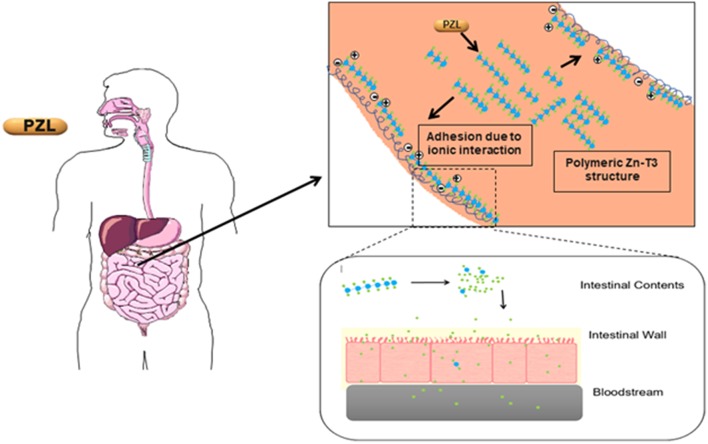
Increasing the period PZL remains at the site of absorption and slowing the rate at which T3 becomes available for absorption, more closely matches T3 production in a healthy subject. By creating a unique “drug depot” from which T3 slowly releases, PZL addresses many of the limitations that have plagued previous attempts to mimic the natural production of T3 in healthy subjects.

Another T3 derivative was prepared using metal coordination, which resulted in LT3 polymers with Zinc. Poly-Zinc-Liothyronine (PZL) is a copolymer of zinc and T3 that forms a supramolecular complex (Figure 2). These supramolecular complexes of the form [M(T3)]_n_ have superior mucoadhesive properties, and when coupled with the hydrolysis behavior of [M(T3)]_n_ complexes, translate into a slow release formulation of LT3 (38). The properties of a controlled release, orally delivered drug product via metal coordination of a drug ligand, relies on known principles of mucoadhesion and coordination chemistry (49). For illustrative purposes only, consider PZL as a prodrug. Then the process of modified drug release and absorption can be seen to involve three distinct steps: (a) Mucoadhesion of PZL to an area of the gastrointestinal tract, (b) Controlled ligand exchange (e.g., hydrolysis) of T3 from PZL, followed by c) Drug absorption of the LT3. Many metal complexes, including PZL, exhibit mucoadhesion due to the interaction of the metal, acting as a Lewis acid, with anionic components of the mucosa (Figure 3) (50). Mucoadhesion prolongs the residence time (i.e., delays transit time) of a drug in the gastrointestinal tract (51). PZL interacts with the mucosa by a variety of additional mechanisms, including coordinate covalent bonding, hydrogen bonding, halogen bonding, metal- halogen bonding, electrostatic interactions and particle size (52–55). Thus, PZL adheres to the intestinal lining creating a “drug depot” from which LT3 gradually releases into the intestinal lumen and is ultimately absorbed into the bloodstream (Figure 4). Intestinal contents, such as bile acids and pancreatic secretion, accelerate breaking of the bonds between the metal and T3. Once freed from the metal, the released T3 reverts to its original form and quickly travels through the epithelial cell layer and into the blood stream. These processes increase the length of time PZL remains in the intestine and slows down the rate at which LT3 becomes available for absorption. Of note, Zn is an essential mineral involved in multiple physiological functions and the amount contained in a 30 ug dose of PZL is <1/1,000 of the daily recommended allowance.

**Figure 5:**
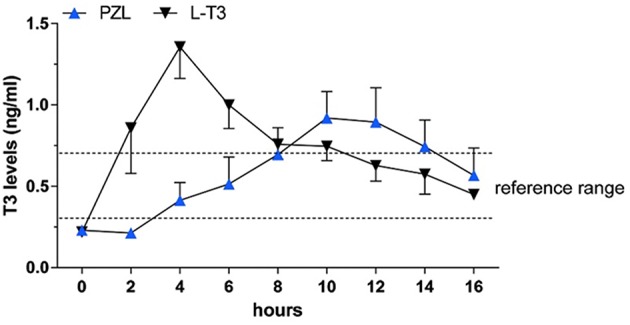
Serum triiodothyronine (T3) levels in hypothyroid rats given 24 μg/kg body weight LT3 or equimolar amounts of PZL through gavage; *adapted fivm* Da Conceicao et al. (38).

Capsules of PZL were tested in hypothyroid rats via oral gavage, with a significant modification in PK properties (38). After PZL administration serum T3 exhibited about 30% lower peak (lower Cmax) that was delayed (longer Tmax) by about 6h as compared to rats given equimolar amounts of LT3. These figures could be further improved by packaging PZL in slow release capsules made with hydrophilic swellable matrix as described above for LT3 (44); the T3 clearance rate did not show differences between PZL- and LT3-treated rats. TSH levels, which were elevated, declined rapidly after LT3 administration but in PZL-treated rats the decline was delayed by about 4 h (Figure 5). Daily administration of PZL for 8 days showed that PZL and LT3 had similar long-term biological effects such as reduction of serum cholesterol, restauration of growth rate and induction of T3-dependent genes in the heart, liver and brain (38).

##### Rectal

The walls of the rectum are lined with a mucosa that is highly vascularized, allowing for rapid absorption of medications. Formal PK studies after the administration of the LT4 suppository have been performed in thyroidectomized rats (56). Tmax is at about 15 h, similar to when LT4 is given orally, but the area under the curve is about 3 times smaller indicating incomplete absorption. Similar results were obtained in hypothyroid patients (56). Unfortunately, no such studies have been performed with LT3 but should be explored.

#### Parenteral Preparations

##### Subcutaneous

One of the most common forms of TH replacement in small rodents is the subcutaneous injection of aqueous solutions of LT3. While this is a very reliable way of delivering exact amounts of LT3, its absorption is relatively fast, followed by a peak of LT3 in the circulation. Peaks can be minimized by splitting the dose into multiple injections every 24 h, but with a half-life of ~2 h, the peaks of T3 in the circulation will inevitably remain (37). A more stable T3 profile could in theory be achieved by injecting an oil suspension of LT3, which should slow down its absorption rate, but formal PK studies remain to be done.

**Figure 6:**
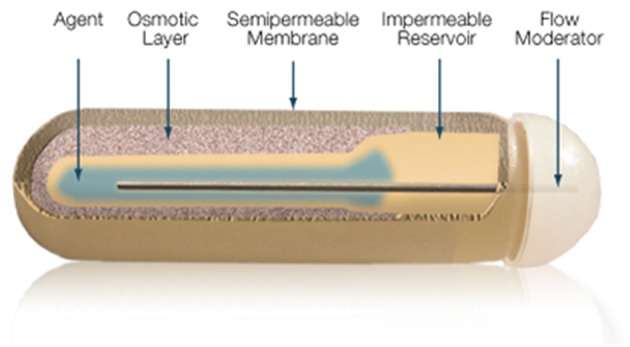
Mechanism of ALZET osmotic pumps which relies on the osmotic pressure difference between pump's compartments. Reproduced from www.Alzet.com.

The gold standard in parenteral preparations for animals is the use of osmotic pumps or pellets that are implanted subcutaneously, releasing fixed amounts of T3 daily for a pre-defined number of days. The pump is a small cylindrical reservoir that uses osmotic driving agent to release a pharmaceutical of interest at a rate of 0.1 to 10 ul/h up to 6 weeks. Both the pumps and the pellets are suitable to be surgically implanted in rodents as well as in larger animals (37). Osmotic minipumps (Alzet, Cupertino CA) operate due to a difference in osmotic pressure between the pump and the subcutaneous area where the pump is implanted. The higher osmolality of the pump flux water through a semipermeable membrane into the pump. As the water builds up into the pump, it compresses the reservoir, displacing the pharmaceutical solution into the subcutaneous at a known rate (Figure 6). The pellet system (Innovative Research of America, Sarasota FL) consists of a pellet with a biodegradable matrix that continuously releases the pharmaceutical product in the animal. Pellets vary in diameter (1/8″ to 1/2″) depending on the pharmaceutical load and release rate. Pellets might contain from 0.001 mg up to 200 mg, which is released over a predefined period of time, e.g., 21 days, 60 days or 90 days. Thyroidectomized rodents implanted with either osmotic pumps or pellet devices do exhibit rather stable levels of T3 in the circulation (37).

Similar technology is being developed by pharmaceutical companies which could provide stable delivery of LT3 to patients and be applicable in the treatment of hypothyroidism. Titan Pharmaceuticals (South San Francisco CA) utilizes a proprietary technology (ProNeura®) to develop a platform to deliver LT3 subcutaneously. It consists of a solid rod made with a mixture of LT3 and ethylene-vinyl acetate that is placed subcutaneously, normally in the inner part of the upper arm, and can be removed at the end of the treatment period. Preliminary studies have been performed in thyroidectomized rats and in intact beagle dogs (57). LT3 implants continuously released T3 in both species, in a dose dependent manner for over 6 months. Following implantation, there was a transient serum peak of T3 followed by a sustained period of relatively stable circulating levels of T3 (57).

MedinCell (Montpellier, France) takes advantage of the fact that copolymers combined with pharmaceuticals are solubilized in a biocompatible solvent, which forms a fully bioresorbable depot once injected subcutaneously. BEPO® consists of copolymers containing hydrophilic blocks (polyethylene glycol—PEG) linked with hydrophobic blocks (Poly(D,L-lactic acid)—PLA), which precipitate and create a depot when placed an aqueous environment. The pharmaceutical is trapped within this matrix and later released by diffusion. The kinetics of pharmaceutical release can be fine-tuned by adjusting the hydrophilicity and relative ratio of the copolymers. Preliminary studies are not available but this technology could also potentially provide a steady release of LT3 molecules for days, weeks or months.

##### Intravenous and intraperitoneal

Both routes have been used to administer LT3 dissolved in aqueous solutions but in both cases a major peak of T3 in the circulation occurs (37).

#### Regenerative Approaches

Thyroid transplant was one of the first approaches to treat hypothyroidism, as many patients improved after receiving animal (sheep or goat) or human thyroid glands (from patients with Graves' disease or goiter) (4). Despite early successes this form of treatment was abandoned as symptoms of hypothyroidism kept recurring. More recently, a method was described that generate thyroid cells from stem cells by overexpressing the transcription factors NKX2.1 and PAX8 in mouse embryonic stem-cells (mESC) (58). This leads to fully differentiated follicular cells that express thyroid-specific markers such as TSH receptor, sodium/iodide symporter NIS and thyroglobulin. When exposed to human TSH, these cells organize with a three dimensional follicular architecture. These structures were next grafted under the kidney capsules of hypothyroid mice to examine their *in vivo* functionality. Serum FT4 became detectable after 4 weeks of transplant along with a reduction in serum TSH levels when compared to levels before grafting (58). Similar experiments were performed by other groups and, as a whole, it is clear that hypothyroid mice can be made euthyroid by transplanting functional thyroid follicular constellations (59). Whether this will ever be feasible to be utilized in humans remains to be seen. Patients complaining of persistent hypothyroidism symptoms could benefit from a new thyroid gland and physiological secretion of both T3 and T4.

#### Tissue Targeting of LT3

Substantial progress on LT3 delivery technology has been achieved recently with the development of tissue-specific targeting of T3 molecules. For example, engineered chemical conjugates of glucagon and T3 enabled delivery of T3 to the liver (60). Treatment with this conjugate corrected hyperlipidemia, steatohepatitis, atherosclerosis, glucose intolerance, and obesity in mouse models of obesity. Liver-directed T3 action spared the cardiovascular system from adverse T3 effects. These findings support the therapeutic utility of integrating T3 and a second hormone into a single molecular entity in order to obtain tissue-specific effects of T3 (60).

Nanotechnology, which involves manipulation of matter sized from 1 to 100 nm, is another tool that has been utilized to achieve tissue-specific delivery of LT3. Nanoparticles containing LT3 have been manufactured using poly(ethylene imine) (PEI) complexed with dodecanoic acid (PEI-C12) with a lamellar nanostructure and a repeat unit of 2.9 nm. In this context PEI-C12 functions as a guest matrix that dissolves LT3 without crystallization (61). This approach was used to test the hypothesis that efficacy of T3 delivery to the brain can be enhanced by encapsulation in nanoparticulate vehicles (62). T3 was encapsulated in poly-(lactide-co-glycolide)-polyethyleneglycol (PLGA-b-PEG) nanoparticles that were coated with glutathione, an efficient means of brain targeted drug delivery. Efficiency of T3 delivery was tested by measuring its ability to protect against ischemic damage in middle cerebral artery occlusion model of ischemic brain stroke. Indeed, administering T3 in nanoparticulate form resulted in significant benefit over injection of a LT3 solution (62).

## Conclusion

LT3 is commercially available as sodium salt packaged in tablets that result in rapid duodenal availability and absorption (Table 1). The development of slow release formulations that could be used in humans has been in the works for years. Claims of slow release LT3 formulations based on changing the composition of the tablets have not been confirmed in clinical trials or have only minimally affected Cmax and Tmax. A number of new strategies and compounds are being explored but details aren't always available due to commercial interests and protection of intellectual property. So far, T3-S and PZL have shown promising results in animal models but formal phase-1 clinical trials have not been conducted. There is no doubt that the momentum around developing new delivery methods of LT3 for humans is building. This will pave the road to better understand and evaluate the use of LT3/LT4 combination therapy and possibly improve patients' quality of life.

**Table 1:** Developmental stage of different LT3 products, their main properties and PK studies in rodents and humans.

**Delivery Route/Formulations**	**Properties of LT3 Preparations**	
	**Humans**	**Rodents**
**ENTERAL**		
Na salt	- PK marked by fast absorption and a 3–4 h peak in serum that subsides after several hours (29)	- Similar PK to humans when given through gavage; mixed with food (38)
Slow release tablet	- Coated tablets; minimal/no PK change (30, 44) - Chewable LT3-resin complex gum; under development	- Not tested - Not tested
Liquid	- Commercially available (41); no PK studies	- Dissolved in drinking water; variable results depending on animal's weight and sex (37)
T3-Sulfate	- Some PK improvement with variable results (47)	- No PK studies; low potency effects (46)
PZL capsule	- Phase 1 clinical trial in 1 year	- Improved PK; similar thyromimetic effects (38)
**PARENTERAL**		
*Subcutaneous*		
Bioresorbable depot	- Under development; steady release for months	- Not tested
Ethylene-Vinyl Acetate Rod	- Not tested	- Relatively stable serum T3 levels for months (57)
Aqueous/oil solutions	- Not used	- Fast absorption with peak of T3 in the circulation
Osmotic pumps/pellets	- Not available in humans	- Stable levels of T3 in the circulation (37)
*Intravenous*	- Immediate peak of T3 in the circulation	- Immediate peak of T3 in the circulation (37)
*Intraperitoneal*	- Not used	- Rapid peak of T3 in the circulation (37)
**REGENERATIVE METHODS**		
Thyroid development from stem cells and transplant	- Not available	- Restored euthyroidism to hypothyroid nude mice; serum T3 levels presumably stable (58)
**TISSUE-T3 TARGETING**		
Hybrid molecules	- Not tested	- Glucagon-T3; no PK studies; liver-specific thyromimetic effects (60)
Nanotechnology	- Not tested	- Nanoparticles containing LT3; no PK studies; brain-specific thyromimetic effects (62)

## Author Contributions

TI performed a bibliographic search and compiled a draft of the manuscript. JP and TP worked on the section on PZL and metal coordination. AB coordinated all work and edited the whole manuscript.

### Conflict of Interest Statement

TI is the President and Chief Science Officer at Synthonics and Adjunct Faculty at Virginia Tech's Department of Chemistry; JP is Vice President of Research and Development at Synthonics; AB is a consultant for Synthonics Inc, Allergan Inc, and BLA Technology LLC; he served as consultant for Sentier LLC during 2018. The remaining author declares that the research was conducted in the absence of any commercial or financial relationships that could be construed as a potential conflict of interest.

## Abbreviations

AUC: area under the curve
Cmax: maximum concentration
D2: type 2 deiodinase
D3: type 3 deiodinase
FT4: free thyroxine
FT3: free triiodothyronine
HR: (place in column) heart rate
HPT: hypothalamus-pituitary-thyroid
LT3: liothyronine
LT4: levothyroxine
mESC: mouse embryonic stem-cells
PZL: Poly-Zinc-Liothyronine
PK: Pharmacokinetics
T3S: T3 sulfate
T3: triiodothyronine
T4: thyroxine
Tmax: time to maximum concentration
TSH: thyroid stimulating hormone.
